# Estimation of ^99^Mo production rates from natural molybdenum in research reactors

**DOI:** 10.1007/s10967-016-5036-6

**Published:** 2016-09-24

**Authors:** M. Blaauw, D. Ridikas, S. Baytelesov, P. S. Bedregal Salas, Y. Chakrova, Cho Eun-Ha, R. Dahalan, A. H. Fortunato, R. Jacimovic, A. Kling, L. Muñoz, N. M. A. Mohamed, D. Párkányi, T. Singh, Van Dong Duong

**Affiliations:** 1 Reactor Institute Delft, Delft University of Technology, Mekelweg 15, 2629 JB Delft, The Netherlands; 2 Division of Physical and Chemical Sciences, Department of Nuclear Sciences and Applications, International Atomic Energy Agency, Vienna, Austria; 3 Institute of Nuclear Physics, Academy of Sciences of Uzbekistan, Ulug Gulomov Str., Tashkent, Uzbekistan 100214; 4 Instituto Peruano de Energia Nuclear, Avenida Canadá 1470, 41, Lima, Peru; 5 Institute of Nuclear Physics of Ministry of Energy of the Republic of Kazakhstan, Ibragimova 1, Almaty, Kazakhstan 050032; 6 RI Research Division, Korea Atomic Energy Research Institute, 989-111, Daedeok-daero, Yuseong-gu, Daejeon 305-353 Korea; 7 Medical Technology Division, Malaysian Nuclear Agency, Bangi, 43000 Kajang, Selangor Malaysia; 8 Instituto Nacionale de Investigciones Nucleares, Carretera Mexico-Toluca S/N La Marquesa, Ocoyoacac, Mexico; 9 Jozef Stefan Institute, Jamova Cesta 39, 1000 Ljubljana, Slovenia; 10 Centro de Ciências e Tecnologias Nucleares and Laboratório de Engenharia Nuclear, Instituto Superior Técnico, Universidade de Lisboa, E.N. 10 ao km 139,7,, 2695-066 Bobadela LRS, Portugal; 11 Comisión Chilena de Energía Nuclear (CCHEN), Avenida Nueva Bilbao No. 12501, Las Condes, Santiago, Chile; 12 Egypt Second Research Reactor (ETRR-2), Atomic Energy Authority (AEA), Abou Zabal, Cairo, 13759 Egypt; 13 Centre for Energy Research, Hungarian Academy of Sciences, Konkoly Thege Miklós út 29-33, Budapest, 1121 Hungary; 14 Department of Atomic Energy (DAE), Bhabha Atomic Research Centre (BARC), Trombay, Mumbai, Maharashtra 400 085 India; 15 Vietnam Atomic Energy Institute, 01 Nguyen Tu Luc, Dalat, Vietnam

**Keywords:** Mo-99, Tc-99 m production, Research reactor utilization, Epithermal neutron self-shielding

## Abstract

Molybdenum-99 is one of the most important radionuclides for medical diagnostics. In 2015, the International Atomic Energy Agency organized a round-robin exercise where the participants measured and calculated specific saturation activities achievable for the ^98^Mo(n,γ)^99^Mo reaction. This reaction is of interest as a means to locally, and on a small scale, produce ^99^Mo from natural molybdenum. The current paper summarises a set of experimental results and reviews the methodology for calculating the corresponding saturation activities. Activation by epithermal neutrons and also epithermal neutron self-shielding are found to be of high importance in this case.

## Introduction

Molybdenum-99 and its daughter nuclide ^99m^Tc are of paramount importance in medical diagnostics: the NRG 2014 annual report of the High Flux Reactor in Petten states that there, about 1/4 of the world’s supply is being produced, and that 1000 people per hour are subjected to diagnostics using Petten-produced ^99^Mo, every hour, every day of the year. At the same time, the ^99^Mo supply is at risk because it is being produced at only a few locations, and mostly as a ^235^U fission product. This has the advantage of very high ^99^Mo specific activity after the chemical separation from the other fission products, but also the disadvantage of generating large amounts of radioactive waste. Recently, the producers are also hampered by the restricted use of highly-enriched uranium targets as an initial material.

The International Atomic Energy Agency (IAEA) [1, 2] and others [3, 4] propose or consider a more distributed network of ^99^Mo production sites, where it would then be produced through the ^98^Mo(n,γ)^99^Mo reaction. The achievable specific activities are less favourable that way, but the absolute amounts can be amply sufficient for local needs. Subsequently, this would also improve the utilization of national research reactors and would ease the issues associated with international imports [5]. Hot-atom chemistry methods have been developed to separate ^99^Mo from the targeted ^98^Mo atoms to improve the specific activity of the product [6].

In 2012, a feasibility study was perfomed at the Atominstitut in Vienna, involving small samples of MoO_3_ [7]. Ryabchikov et al. [8] reported on the complex relationship between the ^99^Mo production rate, the neutron spectrum and neutron self-shielding effects at various resonance energies in natural and enriched Mo, concluding that the use of enriched Mo has a minor effect of the activation rate through the self-shielding of epithermal neutrons by the other Mo isotopes. Both papers stress the dominance of the epithermal contribution to the activation rate. Wolterbeek et al. point out that the epithermal contribution is sometimes overlooked when this production route is compared to others [9].

In Japan, ^99^Mo production from ^98^Mo appears to be well on its way, both solid and dissolved Mo having been investigated as targets [10].

El Abd reports on measurements of the $^{ 9 8} {\text{Mo}}({\text{n}},\gamma )^{ 9 9} {\text{Mo}}$ cross section and resonance integral, performed on metal foils (0.15 mm thick for natural Mo), confirming the generally accepted values [11].

In order to allow the various research reactors in the world to assess their ^99^Mo production capabilities, the IAEA organised a round-robin in 2015 where 16 reactor institutes from all over the globe irradiated sizeable natural molybdenum samples, measured the induced radioactivity and attempted to predict the experimental results quantitatively by performing model calculations. In this paper, the lessons learned are presented, as well as the approximate production capacity of the reactors involved.

## Theory

In the following, often-used existing methods of modelling the neutron activation rate are related to each other. This should make it easy to estimate $^{ 9 8} {\text{Mo}}({\text{n}},\gamma )^{ 9 9} {\text{Mo}}$ production rates, no matter what set of neutron spectrum parameters is available. It is a useful exercise to perform because various definitions of thermal neutron flux (also known as “thermal neutron fluence rate”) and epithermal flux are in use in different places and contexts, and the confusion that might ensue can lead to erroneous production capacity estimates. More in-depth information on the subject and the basic equations given here can be found in [12, 13].

If, in a neutron activation experiments, the neutron flux can be considered homogeneous in the sample, then the activation rate *R* (s^−1^) per atom is given by.

1 $$R = \int\limits_{0}^{\infty } {\varPhi \left( v \right)\sigma \left( v \right){\text{d}}v} = \int\limits_{0}^{\infty } {\varPhi \left( E \right)\sigma \left( E \right){\text{d}}E} ,$$

where *v* is the neutron velocity (ms^−1^), *(v)* is the neutron capture cross-section (m^−2^) for neutrons with velocity *v* and $\varPhi \left( v \right)dv$ is the neutron flux (m^−2^s^−1^) of neutrons with velocities between *v* and $v \, + \, dv$, *E* is the neutron energy (eV), *σ*(*E*) is the neutron capture cross-section (m^−2^) for neutrons with energy *E*, and *∅*(*E*)*dE* is the neutron flux (m^−2^s^−1^) of neutrons with energies between *E* and *E* + *dE*.

If a nuclear reactor is used as the neutron source, it is helpful to distinguish three energy regions: the thermal region (where the neutrons are in thermal equilibrium with the moderator, their velocities are represented by the Maxwell–Boltzmann distribution and the (n,γ) neutron capture cross sections are mostly inversely proportional to the neutron velocity), the epithermal region (where the neutrons are slowing down, the neutron flux is roughly inversely proportional to the neutron energy and the (n,γ) capture cross sections exhibit resonances), and the fast region (where the neutrons have the energy distribution as dictated by the emission of fast neutrons during the ^235^U fission process and the (n,γ) capture cross sections are very small).

The thermal region is taken here to range from 0 to 0.55 eV, where 0.55 eV is the “Cd cut-off energy” and the corresponding neutron flux is then called the “subcadmium flux”, the epithermal region from 0.55 eV to 100 keV as the “epicadmium flux”, and the fast region from 100 keV to several MeV. Equation (1) can then be written as

2 $$R = \int\limits_{0}^{0.55} {\varPhi \left( E \right)\sigma \left( E \right){\text{d}}E} + \int\limits_{0.55}^{{10^{5} }} {\varPhi \left( E \right)\sigma \left( E \right){\text{d}}E} + \int\limits_{{10^{5} }}^{\infty } {\varPhi \left( E \right)\sigma \left( E \right){\text{d}}E} ,$$

Next, it is assumed that the neutron capture cross section in the thermal region can be written as

3 $$\sigma \left( v \right) = \frac{{\sigma_{0} v_{0} }}{v} ,$$

where *v*_*0*_ is 2200 m/s and *σ*_0_ the neutron capture cross section (m^2^) for neutrons of that velocity, and that

4 $$\varPhi_{\text{e}} \left( E \right) = \frac{{\varPhi_{\text{e}} }}{E} ,$$

where *Φ*_e_ is the epithermal flux (m^−2^ s^−1^) at 1 eV. Also, the contribution of the fast neutrons is considered negligible because the (n,γ) capture cross sections are very small for such neutrons. Equation (1) then transforms to

5 $$R = \int\limits_{0}^{10251} {\varPhi \left( v \right)\frac{{\sigma_{0} v_{0} }}{v}{\text{d}}v} + \int\limits_{0.55}^{{10^{5} }} {\frac{{\varPhi_{e} \sigma \left( E \right)}}{E}{\text{d}}E} ,$$

where 10,251 m/s is the neutron velocity corresponding to 0.55 eV. Now, it can be observed that

6 $$n = \int\limits_{0}^{10251} {\frac{\varPhi \left( v \right)}{v}{\text{d}}v},$$

where *n* is the neutron density (m^−3^) in the thermal region, and the conventional thermal neutron flux *Φ*_0_ is defined by

7 $$\varPhi_{0} = nv_{0} .$$

The resonance integral *I*_*0*_ (m^2^) is defined as

8 $$I_{0} = \int\limits_{0.55}^{{10^{5} }} {\frac{\sigma \left( E \right)}{E}{\text{d}}E} .$$

So that Eq. (1) transforms to the Høgdahl [14] convention:

9 $$R = \sigma_{0} \varPhi_{0} + I_{0} \varPhi_{e} .$$

Values for *σ*_0_ and *I*_0_ are tabulated and widely available in the literature [15] as well as in on-line databases.

### Refinements for non-1/v thermal reactions, epithermal activation and self-shielding

In this section, a few refinements often encountered in activation rate modelling are discussed.

If the nucleus that will result from the capture of a thermal neutron has a resonance energy close to the excited state it will be produced in, the capture cross section will not be inversely proportional to the neutron velocity in the thermal range. Then the thermal capture rate *R*_t_ is approximated by

10 $$R_{\text{t}} = g\left( T \right)\sigma_{0} \varPhi_{0} ,$$

where g(*T*) is the Westcott factor, for which tabulated values as a function of the temperature *T* of the Maxwell–Boltzmann neutron velocity distribution are available [16]. Only a few (n,γ)-reactions show this effect to a context-relevant degree in research-reactor conditions. In the neutron activation analysis community, where the highest possible accuracy is of interest, is has been established that the epithermal neutron spectrum shape is better described by

11 $$\varPhi_{\text{e}} \left( E \right) = \frac{{\varPhi_{\text{e}} \left( {E_{1} } \right)}}{{\left( {\frac{E}{{E_{1} }}} \right)^{1 + \alpha } }} ,$$

where *E*_1_ is 1 eV and *α* characterizes the deviation from the perfect epithermal spectrum [12]. Accordingly, the definition of the resonance integral changes to

12 $$I_{0} \left( \alpha \right) = \int\limits_{0.55}^{{10^{5} }} {\frac{\sigma \left( E \right)}{{\left( {\frac{E}{{E_{1} }}} \right)^{1 + \alpha } }}dE} ,$$

which leads to an additional parameter to characterize the dependence of *I*_0_ on α, i.e. the effective resonance energy *E*_r_. This relation can then be expressed as

13 $$I_{ 0} \left( \alpha \right) = \frac{{I_{ 0} - 0.429}}{{E_{\text{r}}^{\alpha } }} + \frac{0.429}{{(2\alpha + 1)0.55^{\alpha } }} .$$

When objects are irradiated that are not transparent to neutrons due to scattering or absorption, the neutron flux inside the material will be affected. This phenomenon is called neutron self-shielding and depends on sample composition, size, and shape as well as on incident neutron energy.

With all these refinements, the sample-volume averaged capture rate is then given by

14 $$R = g\left( T \right)G_{\text{t}} \sigma_{0} \varPhi_{0} + G_{\text{e}} I_{0} \left( \alpha \right)\varPhi_{\text{e}} ,$$

where G_t_ and G_e_ are the thermal and epithermal neutron self-shielding correction factors.

The specific saturation activity *A*_s_ (Bq g^−1^) can then be calculated with

15 $$A_{\text{s}} = \frac{{R\theta N_{\text{A}} }}{M} ,$$

where *θ* is the isotopic abundance, *N*_A_ is Avogadro’s number and *M* is the molar mass of the element.

### Other conventions and neutron spectrum parameters

#### Alternative definitions for thermal or subcadmium flux

In other conventions, similar expressions are derived, but the thermal neutron flux may be defined differently. Beckurts and Wirtz [13], for example, define a thermal flux *Φ*_T_ as

16 $$\varPhi_{T} = n\left\langle v \right\rangle = n\frac{2}{\sqrt \pi }\sqrt {\frac{T}{{T_{0} }}} v_{0},$$

and a corresponding Maxwell–Boltzmann flux-averaged thermal cross-section

17 $$\sigma_{T} = \left\langle \sigma \right\rangle = \frac{{\int\limits_{0}^{\infty } {\varPhi \left( v \right)\sigma \left( v \right)dv} }}{{\int\limits_{0}^{\infty } {\varPhi \left( v \right)dv} }}\frac{\sqrt \pi }{2}\sqrt {\frac{{T_{0} }}{T}} \sigma_{0},$$

where *T* is the temperature associated with the Maxwell–Boltzmann velocity distribution, and *T*_0_ is 293.6 K. In each convention, the definitions of the thermal cross section and the thermal neutron flux match, so the product *R* = *Φσ* always turns out the same. The choice of convention is therefore arbitrary, as long as the corresponding matching pairs of *σ* and *Φ* definitions are used.

Because of the easy availability of literature values for *σ*_0_ and *I*_0_, Eq. (14) with its parameters is taken as the convention to relate the other models to in this paper, in this case by writing

18 $$\varPhi_{0} = \frac{\sqrt \pi }{2}\sqrt {\frac{{T_{0} }}{T}} \varPhi_{\text{T}}.$$

### Alternative definition for epithermal or epicadmium neutron flux

In the reactor physics community, the epithermal or epicadmium neutron flux is often defined as

19 $$\varPhi_{\text{e}}^{*} = \int\limits_{{E_{\hbox{min} } }}^{{E_{\hbox{max} } }} {\varPhi \left( E \right){\text{d}}E} ,$$

where *E*_min_ and *E*_max_ are the energy limits chosen for the integration—various values are used to this end. Using Eq. (4), the two epithermal fluxes are related by

20 $$\varPhi_{\text{e}}^{*} = \int\limits_{{E_{\hbox{min} } }}^{{E_{\hbox{max} } }} {\frac{{\varPhi_{\text{e}} }}{E}{\text{d}}E} = \varPhi_{\text{e}} \left[ {\ln \left( {E_{ \hbox{max} } } \right) - \ln \left( {E_{\hbox{min} } } \right)} \right] ,$$

or

21 $$\begin{aligned} \varPhi_{\text{e}} = \frac{{\varPhi_{\text{e}}^{*} }}{{\ln \left( {\frac{{E_{\hbox{max} } }}{{E_{\hbox{min} } }}} \right)}} \hfill \\ \hfill \\ \end{aligned} .$$

### Alternative spectrum parameters: cadmium ratio, thermal/epithermal ratio

The neutron spectrum shape can be characterized with the parameter *f*, i.e. the thermal-to-epithermal flux ratio, defined as

22 $$f = \frac{{\varPhi_{0} }}{{\varPhi_{\text{e}} }},$$

So that

23 $$\varPhi_{\text{e}} = \frac{{\varPhi_{0} }}{f},$$

and often also with the cadmium ratio *R*_Cd_, i.e., the ratio of the activation rate without and with cadmium cover, defined by

24 $$R_{\text{Cd}} = \frac{{\left( {\sigma_{0} \varPhi_{0} + I_{0} \left( \alpha \right)\varPhi_{\text{e}} } \right)}}{{I_{0} \left( \alpha \right)\varPhi_{\text{e}} }}$$

So that

25 $$\varPhi_{\text{e}} = \varPhi_{0} \frac{{\sigma_{0} }}{{I_{0} \left( \alpha \right)}}\frac{1}{{\left( {R_{\text{Cd}} - 1} \right)}} .$$

Typically, gold is used as the flux monitor and the tabulated values for *σ*_0_ and *I*_0_ for the ^197^Au(n,γ)^198^Au reaction are to be used as a consequence. Imperfect shielding of subcadmium neutrons by the cadmium cover as well as neutron self-shielding in the flux monitor have been disregarded here.

### Determination of neutron spectrum parameters

The neutron spectrum parameters (*Φ*_0_, *Φ*_e_, *f*, α etc.) can be determined in a variety of ways, ranging from theoretical Monte Carlo calculations, where the reactor and the irradiation facility are modelled in their entirety, to experimental irradiation and measurement of appropriate combinations of elements (such as Zr+Au, or Cr+Mo+Au), possibly with and without cadmium cover.

### Neutron self-shielding calculation

Both the thermal and the epithermal neutron flux tend not to be homogeneous throughout the sample. The ratio of the volume-averaged flux within the sample and the flux in the same location in the absence of the sample is the self-shielding correction factor.

In the thermal region, equations to calculate these factors are readily available for various sample shapes. A good overview is given by De Corte [12].

In the epithermal region, the situation is more complex because of the presence of resonance energies where neutron absorption may be extremely high. Two approaches were employed in this work: the MATSSF software developed by Trkov [17] and the method of Martinho [18] and Chilian [19].

## Experimental

The participating countries and reactors are presented in Table 1.

**Table 1:** Participant countries, reactors and their maximum thermal powers

Participant country	Reactor	Power (MW)
Chile	RECH-1	5
Egypt	ETRR-2	22
Hungary	BRR	10
India	DHRUVA	100
Kazakhstan	WWR-K	6
Korea	HANARO	30
Malaysia	RTP	1
Mexico	Triga Mark III	1
Morocco	MA-R1	2
Netherlands	HOR	2
Peru	RP-10	10
Portugal	RPI	1
Romania	Triga II Pitesti	12
Slovenia	Triga Mark II	0.25
Ukraine	WWR-M	8
Uzbekistan	WWR-SM	11
Vietnam	Dalat RR	0.5

The IAEA sent 3 molybdenum samples to each participant in the exercise: approx. 1 g of Mo_2_O_3_ in a polyethylene capsule (9 mm internal diameter × 9 mm height), and two rectangular pieces of Mo metal of 10 × 10 × 1 and 50 × 10 × 1 mm, weighing about 1 and 5 g, respectively.

From the neutron spectrum parameters of the irradiation facility used, as reported by the participants, the capture rates and the specific saturation activities for a very small natural molybdenum sample (i.e. with negligible neutron self-shielding) were calculated, as well as for the actual bulky molybdenum samples. Both epithermal self-shielding correction approaches were applied.

Most participants experimentally activated the three molybdenum samples and measured the induced activities using a (HP)Ge detector.

The irradiation, decay and measurement times were to be chosen so that the precision of the irradiation time would be better than 1 %, the decay time would be long enough to allow the counting of the samples at dead time below 10 %, and the measurement would provide a Poisson uncertainty in the observed peak area of less than 1 %.

The characteristics of the Ge detectors used are shown in Table 2. Full-energy detection efficiency curves were determined with mixed-radionuclide sources emitting multiple gamma-ray energies or combinations of calibrated point sources in all cases, at the same distances used for sample measurements. Each participant used their own, unspecified nuclear data source to this end. All participants used the same gamma-ray yields for the ^99^Mo gamma rays.

**Table 2:** Detector characteristics

Country	Diameter (mm)	Height (mm)	Distance (mm)	Peak efficiency	Remarks(keV)
Egypt	80	100	100	7.81 × 10^−3^	740
				7.60 × 10^−3^	778
Morocco	58.5	76.3	100	1.93 × 10^−3^	
Malaysia	50	20	223	1.54 × 10^−4^	
Vietnam	69.4	67.1	50	1.08 × 10^−2^	140
Hungary	64.9	89.6	300	5.88 × 10^−4^	
Kazachstan	59	50	60	4.94 × 10^−3^	
Netherlands	55	60	150	1.12 × 10^−3^	
Portugal	51	48	295	5.41 × 10^−4^	366
				3.30 × 10^−4^	740
				3.18 × 10^−4^	778
				3.05 × 10^−4^	822
Romania	50	65.4	100	1.74 × 10^−3^	
			150	9.24 × 10^−3^	
			200	5.75 × 10^−3^	
			250	3.90 × 10^−3^	
Slovenia	35.5	50	160	1.88 × 10^−3^	
Ukraine	74	53	1510	3.63 × 10^−5^	778
Chile	49	36	55	2.50 × 10^−3^	
Mexico	42	43.5	57	1.29 × 10^−2^	
			107	3.60 × 10^−3^	
Peru	80.3	54	240	1.58 × 10^−3^	

The detection efficiencies shown apply to the ^99^Mo main energy of 740 keV, if not stated otherwise

From the observed peak areas, the capture rates during activation were calculated (in the process correcting for dead time, coincidence summing effects and counting geometry differences), using

26 $$R = \frac{{\lambda N_{\text{p}} }}{{\left( {1 - e^{{ - \lambda t_{\text{ir}} }} } \right)e^{{ - \lambda t_{\text{d}} }} \left( {1 - e^{{ - \lambda t_{\text{m}} }} } \right)\gamma \varepsilon }}\frac{M}{{wN_{\text{Av}} \theta }},$$

where *N*_p_ is the net peak area after correction for dead time during measurement, *t*_ir_, *t*_d_ and *t*_m_ are irradiation time, decay time and counting time (s), *λ* is the decay rate (s^−1^), *γ* is the gamma mission probability, *ε* is the full-energy detection efficiency and *w* is the sample mass (g).

The detection efficiency for the molybdenum samples differed from the detection efficiencies as measured with small calibration sources, necessitating corrections for gamma-ray self-absorption and geometrical effects. The participants who estimated these corrections used solid angle approaches, where a numerical integration is carried out over the sample volume, taking the pathlength through the sample towards the detector, as well as the distance from the detector, into account at the same time. The result is a single correction factor, rather than two separate ones for geometry and self-absorption. Also, ^99^Mo often emits the 740 keV photon in coincidence with the 181 keV, so that true-coincidence summing corrections must be applied. These correction factors and the dead-time correction factors or methods, if made available by the participant, are shown in Table 3.

**Table 3:** Correction factors as used

Country		Dead time	Gamma self-absorption and geometry	TCC
Egypt	MoO_3_	1.0015	1.0840	1.0000
	1 g	1.0079	1.0720	1.0000
	5 g	1.0020	1.0850	1.0000
Morocco	MoO_3_	1.015		1.007
	1 g	1.022		1.006
	5 g	1.105		1.002
Malaysia	MoO_3_	1	1	1
	1 g	1	1	1
	5 g	1	1	1
Vietnam	MoO_3_		1.28 (140 keV)	
	1 g		1.36 (140 keV)	
	5 g		1.36 (140 keV)	
Hungary	MoO_3_	ZDT	1.0406	1
	1 g	ZDT	1.0020	1
	5 g	ZDT	1.0050	1
Kazachstan	MoO_3_	1.0042	1	
	1 g	1.0063	1.014	
	5 g	1.0383	1.015	
Netherlands	MoO_3_	1.0846	1.0638	1.0050
	1 g	1.0050	1.0395	1.0050
	5 g	1.0299	1.0428	1.0050
Portugal	MoO_3_	LT preset	1.0860	
	1 g	LT preset	1.0360	
	5 g	LT preset	1.0360	
Romania	MoO_3_	Genie	1	1
	1 g	Genie	1	1
	5 g	Genie	1	1
Slovenia	MoO_3_	ZDT	1.1125	1.0078
	1 g	ZDT	1.0262	1.0087
	5 g	ZDT	1.0208	1.0089
Ukraine	MoO_3_	1.0215	1.031	
	1 g	1.0194	1.031	
	5 g	1.0846	1.031	
Chile	MoO_3_	1.0000	1.0000	1.0000
	1 g	1.0000	0.8438	1.0000
	5 g	1.0000	0.9694	1.0000
Mexico	MoO_3_	1.0054		
	1 g	1.0359		
	5 g	1.0460		
Peru	MoO_3_		1.0130	1.0000
	1 g		1.0380	1.0000
	5 g		1.0380	1.0000

All factors are stated so that an uncorrected measured activation rate has to be multiplied with these to obtain the activation rate in a very small sample

*TCC* stands for true coincidence summing, *ZDT* denotes zero-dead time counting

Unity values indicate that the participant deemed the correction negligible, blank values that no data were provided by the participant

The resulting specific Eq. (15). Finally, the measured specific saturation activities were compared to the calculated ones to assess the quality of the various methods used.

During a follow up workshop at the IAEA in Vienna, in December 2015, the participants compared notes, corrected their results for a number of issues found, and decided on the best nuclear data and methods to be used for ^99^Mo production rate estimation. Not all discrepancies were resolved during this meeting—however all results presented in this paper are the ones obtained from the participant-supplied information during or after this meeting, using the agreed-upon methods and nuclear data. The equations used are the ones given above in this paper. The nuclear data used, all taken from De Corte et al. [15], are as shown in Table 4.

**Table 4:** Nuclear data used in this work (1 b = 10^−28^ m^2^)

Reaction	*σ*_0_ (b)	*I*_0_ (b)	*E*_r_ (eV)	*M*	*θ*
^197^Au(n,γ)^198^Au	98.7	1549.6	5.7	196.97	1
^98^Mo(n,γ)^99^Mo	0.131	6.96	241	95.96	0.2413

For the 740 keV gamma-ray of Mo-99, a yield of 12.1 % was used. All data were obtained from [15]

## Results

### Neutron spectrum characterization

In Table 5, the neutron spectrum parameters as reported by the participants are shown (the participants were not instructed on which parameters to supply in the round-robin process). From these parameters, the parameters needed as input for Eq. 14 were calculated using the equations given above as needed. The resulting values are also shown in Table 5. The Westcott factor g(*T*) was assumed to be unity in all cases for the ^98^Mo(n,γ)^99^Mo reaction. In case of unspecified values for the α parameter, a value of 0 was assumed.

**Table 5:** Neutron spectrum parameters and characterization methods as provided by the participants for the various irradiation facilities used

Participant country, facility	Neutron spectrum characterization method	*Φ*_0_ (cm^−2^s^−1^)	*Φ*_e_ (1 eV) (cm^−2^s^−1^)	*Φ*_e_ (integral) (cm^−2^s^−1^)	*E*_min_ (eV)	*E*_max_ (eV)	*α*	*F*	*R*_Cd_	***Saturation activity (Ci/g)***
Chile	Not specified	4.76E+12	4.15E+10							***3.72E02***
Egypt	Au+Zr	2.80E+11	***1.12E+09***				0.180	250		***1.62E−03***
Hungary	Au+Zr	9.37E+13	3.86E+12				−0.038			***1.84E+00***
India	Monte Carlo	3.60E+13	***4.26E+11***	6.00E+12	0.625	8.21E+05				***3.13E−01***
Kazakhstan	Estimated	6.50E+13	***0.00E+00***							***3.47E−01***
Korea	Monte Carlo	6.78E+13	***1.21E+11***	1.45E+12	0.625	1.00E+05				***3.97E−01***
Malaysia	Au, Cd-cover	7.99E+12	***1.54E+11***						4.30	***8.64E−02***
Mexico	Not specified	2.78E+13	***1.56E+11***	1.87E+12						***1.93E−01***
Morocco	Au+Zr, Au+Cr+Mo	6.40E+12	***2.85E+11***				−0.020	22.5		***1.15E−01***
Netherlands BigBeBe	Au+Cr+Mo	2.40E+13	***1.73E+12***				0.000	13.9		***6.18E−01***
Netherlands BP3	Au+Cr+Mo	5.55E+12	***6.97E+10***				0.040	79.65		***4.57E−02***
Peru	Au+Mo+Co+Lu	6.76E+12	1.62E+11				0.071	40		***6.94E−02***
Portugal	Au, Cd-cover	1.90E+12	***1.80E+10***				0.061	42		***1.53E−02***
Romania	Monte Carlo	1.20E+13	***2.88E+11***				0.012	41.65	3.61	***1.41E−01***
Slovenia	Au+Zr, Cd-cover	1.04E+12	3.85E+10				−0.004	27.11		***1.67E−02***
Ukraine	Monte Carlo	1.84E+14	***9.85E+11***						12.9	***1.26E+00***
Uzbekistan	Not specified	3.70E+13	***4.65E+11***					79.65		***3.29E−01***
Vietnam beam	Au+Mo+W, Cd-cover	1.60E+06	***2.43E+02***						420	***8.62E−09***
Vietnam trap	Au+Mo+W, Cd-cover	1.46E+13	1.28E+11				−0.083	114.3	7.29	***1.34E−01***

Values in bold italics were calculated from the other data in the table with the equations given in this paper

Also in Table 5, the specific saturation activities for a very small Mo target are shown, as calculated from the neutron spectrum parameters and the nuclear data for the ^98^Mo(n,γ) reaction, assuming absence of neutron self-shielding, using Eqs. 13, 14 and 15.

Table 6 shows the thermal and epithermal neutron self-shielding factors, the latter as calculated using Trkov’s and Chilian’s methods, for the three sample types.

**Table 6:** Thermal and epithermal neutron self-shielding factors *G*_th_ and *G*_epi_ for the three sample types

	1 g of Mo_2_O_3_ powder		Mo slab 1 × 1 cm		Mo slab 1 × 5 cm	
Method	*G*_th_	*G*_epi_	*G*_th_	*G*_epi_	*G*_th_	*G*_epi_
	0.982		0.954		0.954	
Chilian		0.857		0.742		0.742
Trkov		0.804		0.599		0.574

Two methods were used for the epithermal self-shielding calculation

In Table 7, the measured specific saturation activities are shown, as well as the ratios of these over the values that were calculated with the two epithermal neutron self-shielding methods.

**Table 7:** Measured specific saturation activities as compared to the calculated values obtained with the two methods for epithermal neutron self shielding correction

	Measured saturation activities						Measured over calculated sat.act. Ratios					
Participant country							Trkov			Chilian		
	Mo_2_O_3_	Unc	Slab 1 × 1 cm	Unc	Slab 1 × 5 cm	Unc	Mo_2_O_3_	Slab 1 × 1 cm	Slab 1 × 5 cm	Mo_2_O_3_	Slab 1 × 1 cm	Slab 1 × 5 cm
	Ci/g	%	Ci/g	%	Ci/g	%	Ci/g	Ci/g	Ci/g	Ci/g	Ci/g	Ci/g
Chile	3.21E−02	4.0	3.24E−02	4.0	3.21E−02	4.0	0.931	1.034	1.034	0.915	0.982	0.973
Egypt	1.59E−03	0.3	1.47E−03	0.4	1.49E−03	1.3	1.011	0.977	0.992	1.008	0.966	0.979
Hungary	1.63E+00	0.4	1.45E+00	0.4	1.46E+00	0.4	1.040	1.133	1.172	0.995	0.986	0.993
Kazachstan	6.25E−01	3.0	5.62E−01	2.0	5.79E−01	2.0	1.831	1.695	1.746	1.833	1.697	1.748
Malaysia	6.85E−02	15.0	7.49E−02	5.5	8.27E−02	9.0	0.888	1.118	1.255	0.862	1.023	1.130
Morocco	5.75E−02	0.9	5.32e−02	1.0	4.75E−02	1.0	1.003	1.131	1.034	0.961	0.990	0.883
NL BBB			4.49E−01	2.0	4.42E−01	2.0		1.079	1.094		0.923	0.909
NL BP3	4.27E−02	1.4					1.015			0.996		
Peru	5.46E−02	3.0	5.38E−02	3.0	5.26E−02	3.0	0.877	0.989	0.982	0.853	0.910	0.889
Portugal	1.72E−02	8.0	1.21E−02	8.0	1.32E−02	8.0	1.221	0.949	1.046	1.199	0.898	0.980
Romania	1.04E−01	1.5	1.02E−01	6.0	9.60E−02	1.5	0.833	0.950	0.911	0.807	0.863	0.812
Slovenia	1.45E−02	0.7	1.19E−02	0.6	1.28E−02	0.5	1.004	0.992	1.092	0.965	0.876	0.942
Ukraine	1.55E+00	3.0	1.44E+00	3.0	1.38E+00	3.0	1.305	1.298	1.251	1.290	1.254	1.201
Vietnam beam	7.50E−09	2.0	8.40E−09	2.0	7.30E−09	2.0	0.887	1.024	0.890	0.887	1.024	0.890
Vietnam trap	1.89E−01	2.1	1.85E−01	2.0	1.88E−01	2.1	1.550	1.709	1.760	1.514	1.592	1.618

The 1 s.d. uncertainties were calculated from the Poisson uncertainties in the measured peak areas

## Discussion

Even though many experimental over calculated ratios as reported in Table 7 are close to unity, as they should be, the entire set of results indicates that the calculation and/or measurement of specific saturation activities for a reaction like ^98^Mo(n,γ) is challenging. The contribution of epithermal neutrons to the total activation rate is high—up to 80 % of the total rate in an in-core facility like the BigBeBe in the Netherlands. This, in turn, necessitates accurate epithermal neutron self-shielding corrections.

Disregarding the epithermal contribution entirely leads to gross underestimation of the saturation activity, as occurred in the case of Kazakhstan. In the neutron trap position in the Vietnam Dalat reactor, the epithermal flux was not disregarded but perhaps underestimated in the characterization process.

Not all participants achieved the desired overall precision of 1 % or better in the measured activation rates. In view of the standard deviations of the resulting ratios between measured and predicted activation rates of about 20 % (Kazakhstan excluded), all reported results were still deemed relevant.

In the cases of Malaysia, Peru, Romania and Vietnam (beam position), it appears that confusion may have occurred between the thermal fluxes *Φ*_0_, *Φ*_T_ and the corresponding capture cross sections. It is indeed unfortunate that both *Φ*_0_ and *Φ*_T_ are known by the same name, i.e. “thermal flux”, when the definitions are different by a factor of √*π*/2 = 0.886—a value that appears to occur a number of times as the experimental-over-theoretical ratio in Table 7.

Various participants reported measured specific saturation activities quite close to the theoretical values, like Hungary and Egypt. The Hungarian results strongly suggest that Chilian’s method for epithermal neutron self-shielding correction performs better than Trkov’s. The Egyptian results were obtained with a very thermalized neutron spectrum and do not offer conclusive evidence in this respect. The results from Slovenia and especially The Netherlands, where the least thermal spectrum was employed, suggest that Trkov’s method may overestimate the severity of the epithermal self-shielding, where Chilian’s may underestimate it to a smaller extent.

For neutron spectrum characterization, the Monte Carlo methods used by various participants appear to perform equally well as the experimental methods.

**Fig. 1:**
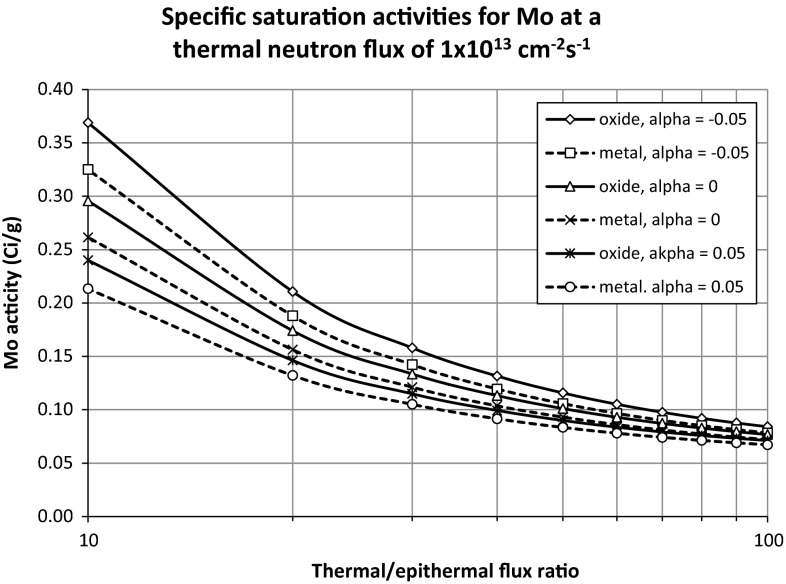
Specific saturation activities to be expected in an irradiation facility with a thermal neutron flux of 1 × 10^13^ cm^−2^s^−1^, as a function of the thermal/epithermal flux ratio, for various values of α and for the two sample types, as calculated with Eqs. 14 and 15 in this paper

To assist the reader in quickly estimating the ^99^Mo production capability in a given irradiation facility, Fig. 1 shows the specific saturation activities to be expected in an irradiation facility with a neutron flux of 10^13^ cm^−2^ s^−1^, as a function of thermal/epithermal flux ratio, for typical values of *α*, molybdenum oxide and 1 mm thick molybdenum metal, as calculated with the procedures and equations (i.e. Eqs. 14, 15) given in this paper, where Chilian’s epithermal neutron self-shielding correction method was used.

## Conclusions

The results presented in this paper demonstrate that the specific saturation activity for the ^98^Mo(n,γ)^99^Mo reaction in natural molybdenum can be estimated with good accuracy, i.e. better than 10 %, if the epithermal activation contribution is taken into account properly and the epithermal neutron self-shielding is corrected for. Both Trkov’s and Chilian’s method give satisfactory results.

In-core irradiation facilities where the epithermal neutrons are strongly present are to be favoured over more thermalized facilities when the goal is to produce as much ^99^Mo as possible.

Metallic molybdenum exhibits more neutron self-shielding than molybdenum oxide, leading to a higher specific saturation activity in the molybdenum itself. On the other hand, due to the lower density, less material can be introduced to the irradiation facility. Finally, post-irradiation processing capabilities available at a given site will determine which material is to be favoured in practice.
